# The impact of psuedouridine modification on human tRNA

**DOI:** 10.1042/BST20250258

**Published:** 2026-06-15

**Authors:** Yasmin Stone, Ethan Morgan, Yong-Han Su, Ching-Ying Kuo, Ting-Yu Lin

**Affiliations:** 1 Centre for Programmable Biological Matter, Department of Biosciences, Durham University, South Road DH1 3LE, U.K.; 2 Department of Clinical Laboratory Sciences and Medical Biotechnology, College of Medicine, National Taiwan University, Taipei, Taiwan

**Keywords:** cancer, neurodevelopmental disorders, Pseudouridine, Pseudouridine synthases, RNA modifications, tRNA

## Abstract

Transfer RNA (tRNA) is an important RNA in cells that decodes messenger RNA (mRNA) codons during protein translation to ensure correct amino acid sequences. The biogenesis of tRNA involves multiple processing steps to produce mature and functional molecules. Pseudouridine (Ψ), a derivative of uridine, is an abundant RNA modification and occurs at multiple positions within tRNAs. These modified sites are highly conserved across organisms. Classical biochemical studies have established that Ψ stabilises RNA–RNA interactions, but structural characterisations and molecular dynamics simulations reveal that Ψ can locally remodel tRNA architecture in ways that are dictated by where it is within tRNAs. Advances in transcriptome-wide Ψ mapping have uncovered additional modified sites beyond those previously described, with several novel sites appearing to be regulated in a cellular context-dependent manner. Furthermore, dysregulated pseudouridylation has been implicated in conditions ranging from cancer to inherited genetic disorders. Together, these developments reframe our understanding of Ψ from a well-established RNA stabiliser to a modification with roles far more dynamic, context-dependent, and clinically relevant than previously appreciated. The present review summarises and discusses the up-to-date developments in the impacts of pseudouridylation on human tRNA biogenesis, tRNA functions, and human health. More mechanistic questions remain open and will require further investigation. As pseudouridylation can also happen in mRNA and rRNA, exploring the interplay between these RNAs will be crucial for fundamental biology and advancing Ψ applications in biotechnology and biomedical uses.

## Introduction

RNA is composed of four basic nucleotides similar to DNA. RNA can be categorised into different classes based on their functions, including transfer RNA (tRNA), messenger RNA (mRNA), ribosomal RNA (rRNA), and many other non-coding RNAs. Although an RNA sequence is faithfully transcribed based on the DNA information, some RNA classes, including tRNA and rRNA, fold into specific shapes. Their signature conformations are evolutionarily conserved across the three kingdoms of life to execute their roles in gene expression and protein translation.

Not only sequence and conformation dictate the RNA function but also RNA modifications contribute to RNA regulation. RNA modifications are chemical decorations that alter the structures of nucleosides, sugar, and phosphate backbones [1]. To date, over 180 classes of RNA modifications have been discovered. Modifications on RNA nucleotides greatly expand their structural diversity, altering their chemical properties. For instance, the added moieties can provide additional hydrogen bonding to facilitate RNA–RNA or RNA–protein interactions. Regulating these interactions is crucial to ensure structural integrity or functional regulation. Disruption of RNA modifications at the transcriptomic level has gained global interest within cellular events associated with health issues, such as ageing [2], metabolic diseases [3], cancers [4–8], and neuron degenerative disorders [9–11]. However, we are far from understanding how chemical modifications impact the function of RNA. The field starts to argue that RNA should have its own counterpart to the Human Genome Project, hence a Human RNome Project has recently launched and aims to unlock the regulatory code of RNA [12].

tRNA was first discovered by Paul Zamecnik and Mahlon Hoagland [13] in 1958. They identified that tRNA can be ‘labelled’ with amino acids and can subsequently transfer the amino acid to the newly synthesised protein polypeptide chain. After the discovery of tRNA, many tRNA chemical modifications were identified by Robert Holley [14] and others (see review [15]), including pseudouridine (Ψ), dihydrouridine, methyladenosine, 5-methylcytidine, wybutosine, and queuosine. Up to now, more than 80 different RNA modifications are found in tRNAs. On average, over 13% of positions in tRNA are chemically modified. These modifications are required for stability and function.

Ψ is one of the most abundant and conserved modifications found in tRNA. In the present review, we will be focusing on recent findings of Ψ in tRNAs and its contributions to tRNA biogenesis, structure, and function.

## tRNA structure, biogenesis, and pseudouridylation

**Figure 1:**
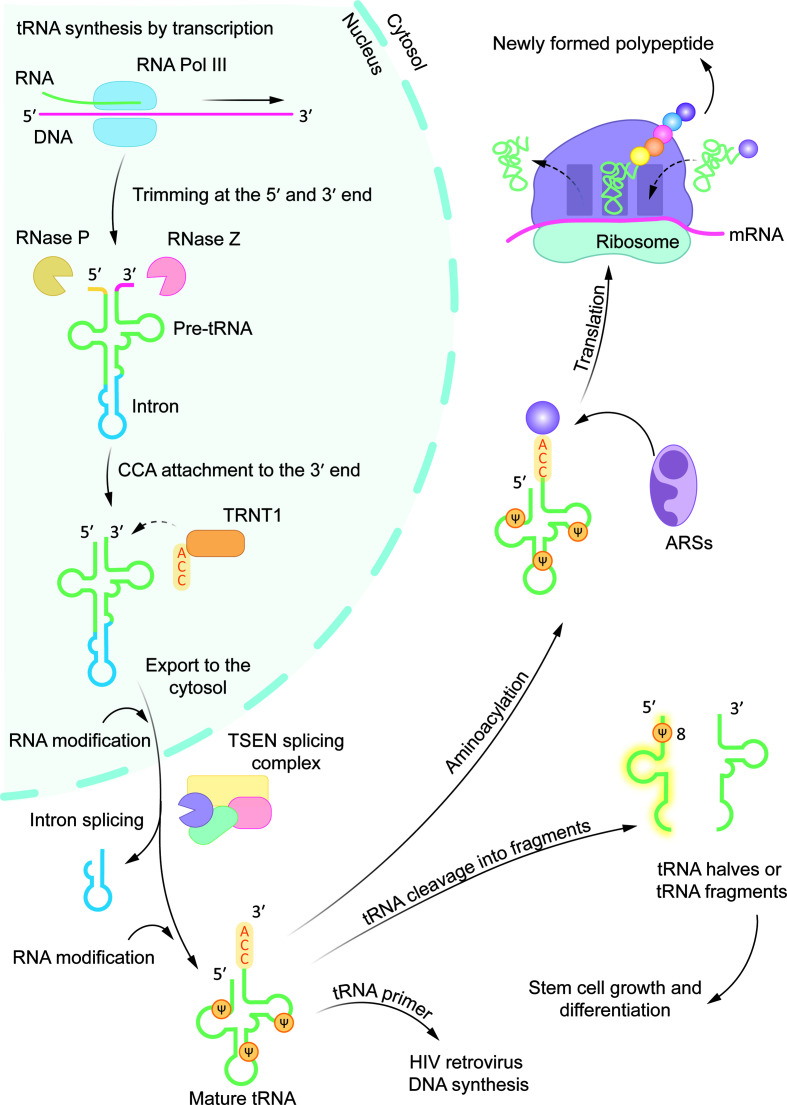
A scheme of tRNA production, maturation, and biological roles tRNA is produced through Pol III-mediated transcription. The produced precursor tRNA (pre-tRNA) is then processed by trimming, intron removal, cytosol exporting, CCA editing, aminoacylation, and RNA modification. Ψs are shown as presented as one type of RNA modification. RNA modification can take place in the nucleus and cytosol. The mature tRNA is then involved in ribosome-dependent polypeptide synthesis, but it can also be processed as tRNA fragments (tRFs) for stem cell regulation or used as primers for HIV DNA synthesis.

tRNA is transcribed as a precursor molecule (pre-tRNA) by RNA polymerase III (Pol III) in the nucleus (see review [16]). The pre-tRNA needs to go through post-transcriptional processing to become a mature tRNA (Figure 1). The following sequential processes include removing nucleotides from both 5′ and 3′ ends, adding an additional nucleotide triplet CCA at the 3′ end, and aminoacylation at the CCA end. Of note, some pre-tRNAs contain introns, which need to be removed through splicing and ligation [17]. Maturation of mitochondrial tRNA (mt-tRNA) follows a similar regulatory process [18]. Chemical modifications in tRNA are part of the maturation process, but at which step these modifications are added remains poorly characterised. Substantial studies using biochemical characterisation confirm many conserved Ψs can be added to mature tRNAs [19–22]. Through direct RNA sequencing targeting the Pol III transcriptome, a proportion of Ψs are added to pre-tRNAs [23].

**Figure 2:**
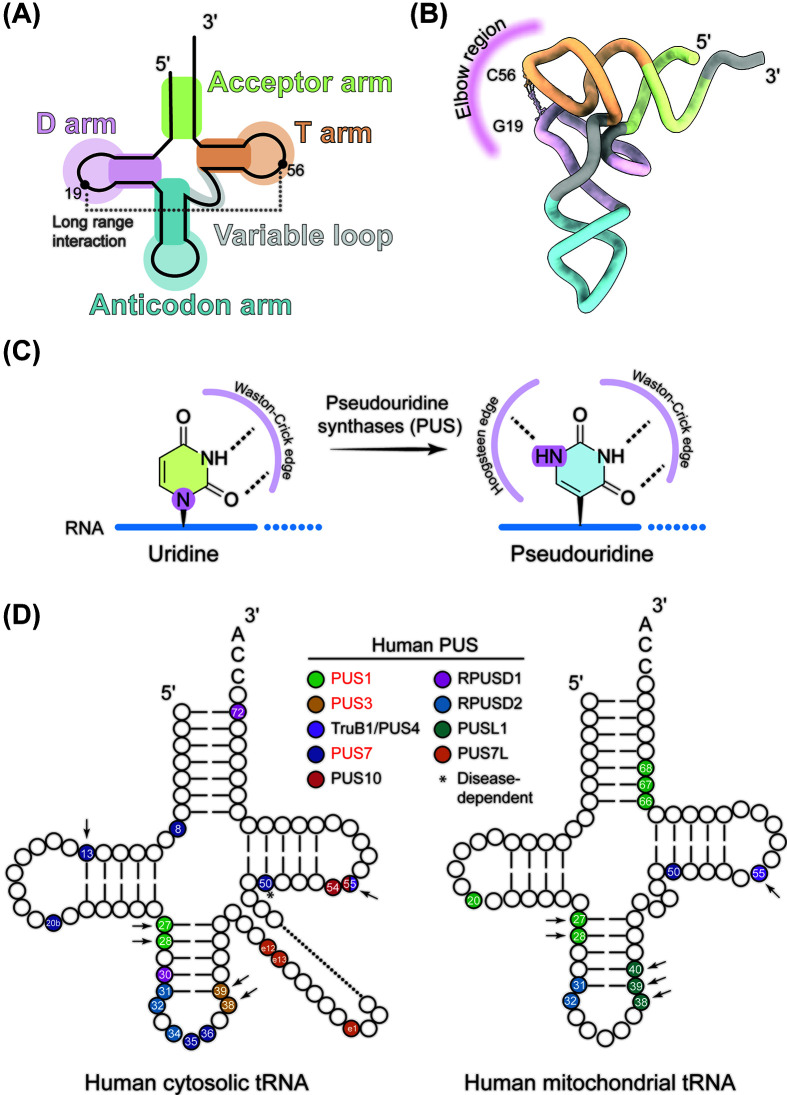
tRNA structures and Ψ landscape in human cytosolic and mt-tRNAs Illustrations of tRNA structures in 2D (**A**) and 3D (**B**). The four arms are indicated: D-arm (purple), anticodon arm (blue), T-arm (orange), and acceptor arm (green). The long-range interaction between D-arm and T-arm forms the elbow region, which is supported by the invariant G19-C56 pair. (**C**) Chemical structures of uridine and Ψ. The hydrogen bonds are indicated as dashed lines, while the Watson–Crick edge and Hoogsteen edge are highlighted. (**D**) Maps of Ψs in human cytosolic (left) and mitochondrial (right) tRNAs. The PUS-dependent modification sites are colour-coded. The positions are highlighted, whereas the disease-dependent Ψ is indicated by *. The highly conserved Ψ sites are indicated by arrows.

There are over 600 tRNA genes (from the Genomic tRNA Database: https://gtrnadb.org/) that consist of 49 isoacceptors in the human genome (termed cytosolic tRNA, cy-tRNA) [24]. In mitochondria, there are 22 mt-tRNAs, which are encoded in the mitochondrial genome [25,26]. tRNA is between 73 and 76 nucleotides (nt) long. Interestingly, while each tRNA has a unique sequence, they (in theory) fold into the same shape. tRNA forms a clover leaf in 2D, consisting of four arms, including the dihydrouracil arm (D-arm, or DHU arm), anticodon stem loop (ASL), TΨC arm (T-arm), and acceptor stem (Figure 2A). The acceptor stem is for conjugating a tRNA-specific amino acid. In 3D, the T-arm and D-arm meet to fold into an ‘elbow’ that forms a signature L-shape (Figure 2B). This particular folding is supported through the continuous stack of bases of A58, G18, G57, G19, and C56, including the Watson–Crick pair between G19 and C56 (see review [27]). The L-shaped structure of tRNA is important as it forms the bridge between mRNA and the production of a protein polypeptide chain. The function of tRNA is to decode mRNA codons through anticodon base-pairing, which in turn directs the incorporation of the correct amino acid, via the charged acceptor arm, into the growing polypeptide chain.

tRNA is heavily modified, especially at the ASL region [28]. In particular, position 34 (a wobble position responsible for pairing to the third codon position in mRNA) is the RNA modification hotspot, which ‘fine-tunes’ the decoding process. This means that the base-pairing at the wobble position affects the tRNA recognition at the ribosome and alters the translation speed [29]. In contrast with the modification at position 34, the remaining modifications are spread across the four arms and are mostly at conserved positions. Ψ, an isomer of uridine, is termed as ‘the fifth nucleotide’ for its high abundance in the overall RNA pool [30]. Chemically, the N1-C1′ glycosidic bond of uridine is cleaved and the base is rotated 180° around the N3-C6 axis (Figure 2C). A new C5-C1′ carbon-carbon bond is then formed between the base and sugar [30,31]. This Ψ structure features an additional hydrogen bond donor at the N1 position, which primarily affects Hoogsteen-based interactions without altering the Watson–Crick face of the base. It means that Ψ can increase the strength and stability through base stacking in a loop (single-stranded structure) or base-pairing within the double-stranded region; see review [32]. Moreover, it can also facilitate interaction with proteins to attenuate activities [33].

This uridine-to-Ψ conversion is called pseudouridylation, which is a conserved enzymatic reaction, mediated by Ψ synthases (PUS) [31]. For detailed information on the PUS-mediated pseudouridylation reactions, we recently have published a review [34]. In cy-tRNA, Ψ is installed at several conserved positions (Figure 2D) [14,32,35], including Ψ13 (PUS7), Ψ27/28 (PUS1), Ψ31/32 (RPUSD2), Ψ38/39 (PUS3), Ψ54 (PUS10), and Ψ55 (PUS10 and TruB1). Likewise, in mt-tRNA, there are known and conserved Ψ positions [26] (Figure 2D). Of note, although these sites are highly conserved, the Ψ pattern is tRNA specific due to sequence diversity [1]. With advances in Ψ detection and mapping, it enables the field to obtain a comprehensive Ψ profile in cy-tRNA [35] and mt-tRNA [26]. In contrast with these conserved Ψ sites, a few additional Ψ sites have been discovered when cells are in different conditions, including Ψ8 found in specific tRNA-derived fragments in stem cell context (PUS7) [36] and Ψ50 in a glioblastoma cell line (PUS7) [8]. In addition, several newly identified Ψ sites have been reported [35], such as PUS7-dependent sites (e.g. 20b, e12, and e13) and RPUSD2-dependent sites (e.g. Ψ35 in cy-tRNA^Ile^). However, biochemical characterisations are still required to provide explanations on how PUS achieves such ‘dynamic’ activity regulation.

## Impacts on stability and structure

All tRNA, including class I, class II cy-tRNA, and mt-tRNA, form a conserved L-shaped structure, which has been confirmed by various crystal structures [37] [i.e. yeast tRNA^Phe^ (PDB 1EHZ) or human tRNA^Sec^ (PDB 3A3A)]. Structures determined through crystallography display the most stable form, and the question is whether tRNA has the same form in solution. A recent study has determined eight human unmodified cy-tRNA cryo electron microscopy (cryoEM) structures and presented the conserved L-shaped conformation [19]. In addition, two cryoEM structures of endogenous human tRNA (i.e. tRNA^Lys^ and tRNA^Arg^) extracted from cell lines demonstrated that the overall L-shape is still retained in solution despite the full modifications [19].

To dissect Ψ in structure stability attenuation, a recent study took on a systematic approach to measure the impact of four conserved Ψs (i.e. Ψ13, Ψ27/28, Ψ38/39/40, and Ψ55) in tRNA stability and conformation (Figure 2D). When a tRNA is fully modified with its conserved Ψ sites, overall thermostability increases [19]. This enhanced stability also reflects on the cryoEM structure resolution improvement, especially at the core region. The pronounced stabilising effect of Ψ13 and Ψ55 likely arises because the tRNA core is formed through tertiary interactions between the D-arm and T-arm. Molecular dynamics simulations of tRNA folding [19] further suggest that this D–T-arm interaction represents a structural vulnerability, an ‘Achilles’ heel’, that must be stabilised to ensure proper tertiary structure formation. The effect is also conserved in Ψ55 in human mt-tRNAs, including mt-tRNA^Asn^, mt-tRNA^Gln^, mt-tRNA^Glu^, and mt-tRNA^Pro^, which governs the crucial base-pairing (18A/G-Ψ55) for securing the structure [38]. This effect was observed in mt-tRNA^Glu^, which has an altered conformation and reduced stability and abundance due to the loss of Ψ55 [39].

In contrast with the pseudouridylation in the core region, Ψs locating at distal regions, such as ASL for Ψ27/28 or Ψ38/39, generally have a limited impact on overall tRNA tertiary stability [19]. Biochemical characterisations on Ψ38/39 within human cy-tRNA^His^, cy-tRNA^Lys3^, and yeast tRNA^Phe^ show increased local thermostability of the stem-loop structure through stabilising and base-stacking within the 31–39 base-pair [40–42]. Whether Ψ31/32, at the stem region of ASL, have similar effects, it still remains to be investigated.

## Impacts on tRNA biogenesis and function

As mentioned earlier, tRNA maturation requires multiple processes to ensure proper structure and function. Pseudouridylation is part of the tRNA modification landscape. Despite the roles of Ψ in tRNA stability and local structure folding, the presence of Ψ seems not required to prime other RNA modifications [19,43] (Table 1). Although a recent Ψ mapping study proposes there could be a cross-talk of pseudouridylation network in tRNA ASL region [44]. To date, there is no other strong evidence about how the pseudouridylation status in human tRNA affects the tRNA abundance [45], aminoacylation [45], or other RNA modification levels [35,43,46]. However, a recent study found that the loss of Ψ27/28 in cy-tRNAs and mt-tRNAs alters mt-tRNA (i.e. mt-tRNA^Cys^, mt-tRNA^Ser(UCN)^, mt-tRNA^Ala^, mt-tRNA^Tyr^, mt-tRNA^Gln^, mt-tRNA^Tyr^, and mt-tRNA^Ile^) and cy-tRNA profiles [47]. In particular, loss of Ψ27/28 affects translation control of specific transcripts that are responsible for respiratory chain complexe formation and results in mitochondrial dysfunction [47]. Of note, the mt-tRNA^Ala^ and mt-tRNA^Gln^ do not contain Ψ28, and this abnormal expression level could be due to a secondary effect from the perturbed tRNA expression profiles.

**Table 1:** The list of pseudouridine sites in human tRNAs and their biological roles and disease relevance

Site	Enzyme	Biochemical characterised	Biological roles	Disease association
**Core (T-arm and D-arm)**				
13	PUS7	Yes [19,43]	Stabilises D-arm/T-arm interface and increases tRNA thermostability [19]	Neurodevelopmental disorders [43,67–72]
20b	PUS7	No [35]	Unknown	Unknown
20	PUS1 (mitochondria)	No [26,35]	Unknown	Unknown
25	PUS1 (mitochondria)	No [26,35]	Unknown	Unknown
54	PUS10	Yes [19]	Unknown	Unknown
55	TRUB1 (cytosol/mitochondria)/TRUB2 (mitochondria) & PUS10	Yes [19,38,39]	Secures 18A/G-Ψ55 tertiary interaction [38]	mt-tRNA mutation Diabetes/deafness [39]
**Anticodon stem-loop**				
27/28	PUS1	Yes [19]	Contributes to some tRNA abundance [48]	MLASA Mitochondrial dysfunction [79, 81, 82]
30	RPUSD1	No [26,35]	Unknown	Unknown
31/32	RPUSD2 (cytosol and mitochondria)	No [35]	Unknown	Unknown
34	RPUSD2	No [35]	Unknown	Unknown
35	PUS7	Yes [35]	Decoding [50]	Unknown
36	PUS7	No [35]	Unknown	Unknown
38/39	PUS3	Yes [9,19,46]	Enhances base stacking Stabilises codon–anticodon helix [40–42]	Intellectual disability [9,60–65]
38/39/40	PUSL1 (mitochondria)	No [35]	Unknown	Unknown
Variable loop				
e12/e13/e1	PUS7L	No [35]	Unknown	Unknown
Acceptor arm				
8	PUS7	No [37]	Mediates protein translation through tRNA halves [37]	Stem cell defects Myelodysplastic syndromes [37]
50	PUS7	No [8]	Alters ASL geometry Codon-biased translation [8]	Glioblastoma [8]
66/67/68	PUS1 (mitochondria)	No [26,35]	Unknown	Unknown
72	RPUSD1	No [35]	Unknown	Unknown

The T-arm in tRNAs, in both cy-tRNA and mt-tRNA, contains a highly conserved Ψ55, while some have both Ψ54 and Ψ55. Modification in the T-arm in bacterial tRNA (i.e. Ψ55 and 5-methyluridine 54) increases tRNA folding, modification, and global aminoacylation in all tRNAs [48]. However, this regulation does not appear to be a critical criterion in human tRNAs [38]. Disruption of the Ψ55 modification in humans has shown no effect on the quantity of some mt-tRNAs or their aminoacylation status (i.e. tRNA^Asn^, tRNA^Gln^, tRNA^Glu^, and tRNA^Pro^). Human tRNA^Lys3^ contains Ψ54 and acts as primers in retroviral DNA synthesis [49] (Figure 1). Whether Ψ54 in this tRNA contributes to the fidelity and efficacy during HIV reverse transcription still needs to be investigated.

tRNA function is to decode mRNA through base-pairing between the anticodon (tRNA, positions 34, 35, and 36) and the codon (mRNA). Ψ at this anticodon region is found [26,35,50] in human tRNA^Ile^, tRNA^Tyr^, tRNA^Arg^, and mt-tRNA^His^. While there is no evidence on the role of Ψs in anticodon–codon base-pairing in these human tRNAs, mechanistic studies in other organisms have been performed. The tRNA^Ile^ and tRNA^Tyr^ in yeast [51] or *Nicotiana rustica* utilise Ψs in the anticodon region for stabilising anticodon–codon pairing for translational fidelity [52,53]. Notably, a tRNA^Tyr^ gene contains an intron that is required for the Ψ35 installation. However, the presence of Ψ35 is not a pre-requisite for the splicing of the premature tRNA^Tyr^ in plants [54]. In contrast with modifications that directly participate in decoding, Ψ38/39 in bacteria and yeast tRNAs contribute to maintaining codon–anticodon helix stability during translation at the ribosome A site (see review [55]). These modifications support proper triplet conformation, particularly in AU-rich or weakly paired codon–anticodon interactions and in cases involving non-Watson–Crick base-pairing.

Pseudouridylation in U50 has recently been discovered in the tRNA^Arg(CCG2-1)^ from a human glioblastoma stem cell line [8] (Figure 2D). This uncommon position modification is speculated as a result of higher pseudouridylation activity from the overexpression of the PUS7 protein. This pathogenic-relevant Ψ50 in tRNA^Arg(CCG2-1)^ displays a selective translational inhibition towards transcripts enriched for the cognate codon. This is also confirmed by the altered occupancy in ribosome profiling of the codon-specific transcripts. However, the mechanistic properties of the Ψ50’s role in tRNA^Arg(CCG2-1)^ still remain to be investigated (Table 1).

Advances in Ψ mapping discovered PUS7-dependent Ψ8 in specific cleaved tRNA products [36] from tRNA^Ala^, tRNA^Cys^, and tRNA^Val^. These cleaved products are between 18 and 22 nt, which are not classic tRNA fragments (tRFs) but mini tRFs. These mini tRFs contain a guanine-rich sequence located at the 5′ end (>4Gs), which forms a stable tetramolecular RNA G-quadruplex structure and is functional in mRNA translation [56]. The G-quadruplex structure is important for tRFs selectively binding to polyadenylate-binding protein 1, a central initiation factor, repressing protein synthesis through destabilising the eIF4F complex. This regulatory mechanism is important to govern stem cell growth and differentiation in human embryonic stem cell lines [36]. Similarly, this axis of PUS7-Ψ8-mini tRFs also contributes to hematopoietic stem cell function. Despite the important role in early cell differentiational control, we are still not clear how Ψ8 contributes to tRF-mediating mRNA translation and how PUS7 executes Ψ8 formation in tRNAs [20].

## Impacts on human diseases

Early insights into Ψ biology were largely derived from studies in bacteria model organisms. Disruption of pseudouridylation processes is not lethal but reduces growth or fitness in bacteria [57,58] and yeast [59]. In stark contrast, accumulating clinical case studies links disruption of pseudouridylation to a wide spectrum of pathological symptoms in various diseases, in particular neuron-related disorders [9,39,43,60–71] (also see review [72]). One plausible explanation is that neurons are particularly vulnerable to impaired tRNA modification because they depend heavily on accurate and efficient translation for protein synthesis [73]. This has been observed in many other abnormalities in tRNA modification processes [10,74]. To our knowledge, the majority of case studies on defects in pseudouridylation state that they are caused by abnormal enzyme activities [32,34]. In contrast with various mutations reported in the RNA modification enzyme genes, mutations in tRNAs rarely result in diseases. This is due to genome-encoded tRNA genes often being redundant (e.g. isodecoder and gene duplication) that buffers the effect of point mutations in a single tRNA gene. To date, only one case study reports on mutations in U55 of mt-tRNA [39]. The inherited mutation in U55 in mt-tRNA^Glu^ (homoplasmic m.14692A→G) is found in patients with inherited diabetes and deafness. Using lymphoblastoid cell lines derived from the patients, the study reported the destabilised mt-tRNA^Glu^ conformation and defective respiratory capacity resulting from the reduced translation of mitochondrial complexes I and IV [39].

As some human PUS, such as PUS1 and PUS7 [43], modify both tRNA and mRNA [75,76], it is difficult to deconvolute the specific impact of tRNA pseudouridylation on disease onset. Nonetheless, studies on PUS3 can provide a clear view because PUS3 modifies tRNA only [46]. It has been shown that PUS3-dependent Ψ38/39 in tRNA attenuates protein translation regulation in yeast, whereas there is no direct biochemical evidence of translational defects in a human system. Patients with complete loss of PUS3 activity indeed display neurodevelopmental disorders, epileptic encephalopathy, and multiple symptoms with cognitive disorders and intellectual disabilities [9,60–65] (Table 1). Similarly, loss of function in PUS7 has been linked to neuron-related disorders [43,66–71], whereas PUS1 deficiency is correlated to anaemia [77] and an oxidative phosphorylation disorder [78,79] called mitochondrial myopathy with lactic acidosis, and sideroblastic anaemia (MLASA). PUS7 is responsible for Ψ13 in many tRNAs [43], Ψ35 in pre-tRNA^Tyr^ as well as in mRNA [80], thereby influencing a wide range of cellular functions. Specifically, loss of PUS7-dependent pseudouridylation activity leads to apoptosis [80] and behavioural changes in a *Drosophila* model [43] and in some clinical cases [43,66,69,70]. Moreover, a survey of human cancers linked loss of PUS7, likely through the PUS7-Ψ8-mini tRFs axis, to haematological malignancies in myelodysplastic syndromes [36]. In addition, overexpression of PUS7 has been found to promote cancer progression [4,8]. As mentioned earlier, PUS7 is linked to an addition of Ψ50 in tRNA^Arg(CCG-2-1)^ in glioblastoma [8]. The underlying pathological mechanism may be attributable to pseudouridylation of tRNA and/or mRNA and warrants further investigation. PUS1 mediates Ψ27/28 in many cy-tRNA and mt-tRNAs and mitochondrial mRNAs. This may also explain how defects of PUS1 result in mitochondrial redox system dysfunction [81,82], with pronounced effects in muscle [82] and erythropoiesis [47,83]. While the link between several tRNA pseudouridylations and diseases has been established, it is still unclear whether other known tRNA-targeting PUS, including TruB1, TruB2, PUS10, and PUSL1, contribute to disease onset or progression.

## Applications and future perspectives

We have summarised impacts of Ψ on tRNA biogenesis, modification cross-talk, and human health. tRNA not only holds a crucial role in protein synthesis for fundamental biology but also now has been utilised in various scientific applications. For instance, synthetic tRNAs are employed to incorporate exogenous amino acids for orthogonal protein synthesis for customised protein properties [84]. tRNA therapeutics have used to treat genetic diseases [85,86] while tRNA modification profiles are proposed to be used for disease diagnostic tools [87]. Therefore, we envision the tRNA field will continue to expand, and we will learn new biological functions and applications. We would like to highlight there are tRNA-like structures, called MALAT1-associated small cytoplasmic RNA and MEN β tRNA‐like small RNA, which are processed from long non-coding RNA metastasis-associated lung adenocarcinoma transcript 1 (MALAT1) [88] and MEN β, respectively. These tRNA mimicries are structurally similar to canonical tRNA, but it is not clear whether pseudouridylation also takes place on such motifs and requires further investigation.

Ψ is regarded as a ‘silent’ modification. Unlike other RNA modifications, it was difficult to detect because Ψ does not alter the Watson–Crick interface to cause modification-induced mismatches during reverse transcription, and it shares the same molecular weight as a uridine, which is indistinguishable for mass spectrometry-based detection. Over the past decade, efforts have been made to develop Ψ-seq methods that introduce modification-induced mismatches or stops during reverse transcription [89–92]. Recently, direct RNA sequencing by Oxford Nanopore Technologies [93,94], which captures ionic current signals as RNA strands translocate through the nanopore, appears to be particularly well suited for this task. Thanks to advancements in RNA Ψ detection at the transcriptome level (see review [95]), the field starts to realise Ψ can control translation through pseudouridylation in mRNA. Ψs in mRNA are found across untranslated regions and coding regions [32]. They affect decoding, translation fidelity, translational speed, and readthrough premature stop codons [96]. Such modifications, unlike the static pseudouridylation in tRNA, can be triggered by external stimulation for cells to adapt to stress [94,97–101]. This regulation empowers the roles of Ψ in gene expression and protein translation, demonstrating a fundamental role in the rule of life.

The strategic incorporation of Ψ and its derivative N1-methylpseudouridine into synthetic RNAs, including mRNA [102] and tRNA [103], has transformed RNA biotechnology and therapeutic development [104,105]. These modifications enhance RNA stability [106,107], increase translational efficiency [107–109], and attenuate innate immune recognition [103,110]. For regulating immune responses, Ψ in RNA is recognised via TLR7 and TLR8 as an essential cell self-defence mechanism to distinguish nonself-derived RNA species [111]. This enables the widespread application of synthetic mRNAs and engineered tRNAs in clinical settings. Beyond passive incorporation, programmable pseudouridylation has also emerged as an active RNA-engineering strategy, enabling targeted modulation of RNA structure and function [112]. The RESTART platform engineers RNA pseudouridylation through NAP57 and a guide RNA system to modify site-specific uridines, which suppresses premature termination codons, restoring protein expression in a disease-relevant context. This strategy has been successfully demonstrated in modulating the efficacy in hereditary deafness within mice [113].

We believe the field will benefit from quantitative detection of Ψ across transcriptomes, which may enable biomarker discovery and customised RNA design with functional modularity. As computational models for Ψ site prediction mature [114,115], principles learned from tRNA pseudouridylation are transferable to mRNA or other RNA engineering pipelines.

## Perspectives

- Ψ modifications in tRNA contribute to structural stability, influence tRNA folding, and translational fidelity in a site- and context-dependent manner.
- Advances in Ψ detection methods have revealed that tRNA pseudouridylation is not uniformly static; some sites are constitutively modified, while others are dynamically regulated, adding an underappreciated layer of post-transcriptional control.
- Ψs in tRNA exert distinct biological effects on tRNA function, biogenesis, and associated disease pathways. Understanding these principles will be essential for deciphering the functional significance of pseudouridylation in mRNA and other RNA species.

## Open Access

Open access for this article was enabled by the participation of the University of Durham in an all-inclusive Read & Publish agreement with Portland Press and the Biochemical Society under an agreement with JISC.

## Abbreviations

Ψ: pseudouridine
ASL: anticodon stem loop
cryoEM: cryo electron microscopy
cy-tRNA: cytosolic tRNA
D-arm: dihydrouracil arm
MALAT1: metastasis-associated lung adenocarcinoma transcript 1
MLASA: mitochondrial myopathy with lactic acidosis, and sideroblastic anaemia
mRNA: messenger RNA
mt-tRNA: mitochondrial tRNA
pre-tRNA: precursor tRNA
Pol III: RNA polymerase III
PUS: pseudouridine synthase
rRNA: ribosomal RNA
T-arm: TΨC arm
tRNA: Transfer RNA
tRF: tRNA fragment
